# Remember the Poor

**Published:** 1912-12-21

**Authors:** 


The Hospital

The Workers' Newspaper of Administrative Medicine and Institutional Life, Administration, National Insurance and Health.

No. 1381, Vol. LIII. SATURDAY, DECEMBER 21, 1912. PRICE ONE PENNY.

### REMEMBER THE POOR.

Many years ago one of the most philanthropic members of probably the wealthiest family in the world was standing at his mother's death-bed. She had been a splendid mother to her sons, and her last words were: "Remember the poor." Those words were written on the memory of her son and became the guiding influence of his life. We have long enjoyed the pleasure of this gentleman's friendship and of his co-operation in many good works. We can testify from personal knowledge that there has probably never been any poor person in need of help, or counsel, or money, who deserved it, that has not had all three in full measure, in memory of a mother who was one of the best of women.

Charles Dickens has immortalised a character which, in his portrayal, was made to represent the exact opposite to the rule of life here recorded. But a man may be prodigal in gifts out of his material resources and yet be Scrooge-like in the influence which he may exercise in his day. This Scrooge-like line of conduct in public affairs seems to us to become more and more apparent in politics since party has been exalted to the first position and statesmanship has been relegated to the last. It is one of the ironies of fate that the Mother of Parliaments, where the members formerly were entitled to be regarded as legislators, is to-day an assembly where real legislation has become impossible. This irony is emphasised and driven home by the circumstance that the chief measures admitted to its consideration purport to improve, do, in fact, affect seriously the social welfare and well-being of the people. The speeches of politicians declare in words that their Acts are conferring benefits upon the humbler classes without the taint of charity, will add to their resources and make their homes and lives brighter and easier than ever before. These undiscussed measures forced through Parliament by mechanical majorities of persons who, for the most part, display little or no interest in them, are turning out in practice to be for the humbler classes, and especially for the very poor, sometimes burdensome and often cruel. We feel, as every member of the community must feel who, like ourselves, is not in politics, that unless individual members of Parliament combine in large numbers to protect the poor they will, in fact, go down to history as a modern type of Scrooge, which added immeasurable misery to infinitely greater numbers of people, than anything that all the Dickens type of Scrooges in the world, however wealthy, have ever produced.

It may be felt by some that thoughts like these are hardly proper reading for Christmastide 1912. Our columns to-day, however, contain evidence that through misapprehension and want of knowledge relates to matters which the nation as a whole must earnestly consider, and combine to face, for the sake of the poor. We wonder how many men and women in the British Islands have yet realised how burdensome may prove the compulsory payment of 3*d.* a week out of a total earning power of 7*s.* Some of the clergy have given us instances where this weekly 3*d.* has deprived the worker of the power to continue a little material comfort without which her vitality may be diminished to the vanishing point. Our voluntary hospitals stand with their doors wide open for the admission of every urgent medical and accident case during each twenty-four hours, but their resources, great as they are, are strictly limited, and are liable to be quickly diminished when taxation grows burdensome, and the pressure upon relatively small incomes increases by leaps and bounds. This state of affairs exists in many middle-class homes at the present time, and our observation and inquiries go to show that they shelter the very persons who in large numbers form the backbone of our hospitals, because it is to them we owe the hundreds of thousands of guinea and half-guinea subscriptions and donations. In another column will be found a weighty speech by the Speaker of the House of Commons which gives striking evidence that the pinch which individual hospitals are feeling, the King's Fund has experienced to a serious extent during 1912.

We wish all workers in hospitals a Merry Christmas; but the outlook for 1913, so far as the managers are concerned, has never been so anxious. Our columns this week will enable our readers to learn why this grave state of affairs in the voluntary hospital world has arisen and must be faced. There is, however, a brighter side to everything, and this is the side which will be presented by the voluntary hospitals on the 25th inst. Hospitals will be lightened this Christmas by the faith which will find expression within their walls on Christmas Day. Whatever individuals may or may not do, each voluntary hospital at any rate always remembers the poor. It is not with them a question merely of money value or physical benefit, but one of personal endeavour and self-denying exertion to make Christmas Day a day of joy and inspiration for every patient. Those who then give of themselves in whole-hearted personal service will, we know, be inspirited and helped to higher ideals and a better life through the work they will do for others. For the outside world assembled in family gatherings, when young and old unite in sweet companionship and renew happy memories though absent from the hospital wards, should remember the sick and take this opportunity, as the members of our Royal Family invariably do, to afford every member of each household an opportunity to contribute something towards the League of Mercy, or to the support of some hospital. Let everybody everywhere, as an act of praise to the great Giver of all good, for the blessings of health, combine in a special family effort on Christmas Day, and give with deliberate generosity. Then these family offerings will at any rate be worthy of the occasion, for every grateful heart cannot fail to remember the claims of the poor, and all should be strengthened by the remembrance.

